# Atypical Intra-articular Osteoid Osteoma Mimicking Slipped Capital Femoral Epiphysis: A Rare Diagnostic and Therapeutic Challenge in a Pediatric Patient

**DOI:** 10.7759/cureus.87493

**Published:** 2025-07-07

**Authors:** Kaan Sevgi, Sinan Nazif Aran

**Affiliations:** 1 School of Medicine, Kansas City University of Medicine and Biosciences, Kansas City, USA; 2 Department of Family Medicine, Demiroglu Science University, Istanbul, TUR

**Keywords:** advanced imaging, ct scan, lytic lesion, mri, osteoid osteoma, pediatric hip pain, radiofrequency ablation, rfa, scfe, slipped capital femoral epiphysis

## Abstract

This case report describes a complex diagnostic challenge in a 10-year-old male presenting with persistent and progressively worsening hip pain. The initial clinical suspicion centered on slipped capital femoral epiphysis (SCFE), a common orthopedic condition in adolescents often associated with obesity and characterized by displacement of the femoral head at the growth plate. Standard radiographs suggested potential epiphyseal abnormalities, raising concerns for SCFE. However, subsequent advanced imaging revealed a 1 cm lytic lesion in the femoral neck with surrounding sclerosis, a classic radiologic feature of osteoid osteoma, thereby confirming the diagnosis.

The patient underwent successful treatment with CT-guided radiofrequency ablation (RFA) with adjunctive perilesional cryotherapy and intraoperative nonsteroidal anti-inflammatory drug (NSAID) infusion, resulting in rapid pain relief and expedited recovery. This case underscores the diagnostic overlap between SCFE and osteoid osteoma, as both conditions can manifest with hip pain and restricted range of motion in pediatric patients. It emphasizes the critical role of advanced imaging modalities, such as magnetic resonance imaging (MRI) and computed tomography (CT), in distinguishing between these two entities, particularly when initial radiographs are inconclusive.

## Introduction

Slipped capital femoral epiphysis (SCFE) is a significant orthopedic condition in adolescents, characterized by displacement of the femoral head at the growth plate, often due to mechanical and endocrine factors [1]. Obesity, trauma, and hormonal imbalances are well-established contributors [2]. Obesity increases axial load on the physis, predisposing to slippage, while endocrine disorders such as hypothyroidism and renal osteodystrophy may weaken physeal cartilage [3].

Clinically, SCFE presents with progressive hip, thigh, or knee pain and a characteristic antalgic gait with external rotation [1]. Early recognition and stabilization are critical to prevent complications like avascular necrosis and chondrolysis [2].

Osteoid osteoma, a benign osteogenic tumor common in adolescents, causes localized, often nocturnal, pain alleviated by nonsteroidal anti-inflammatory drugs (NSAIDs) due to prostaglandin production [4,5]. When intra-articular, it may present atypically, mimicking joint pathologies including SCFE [6-8]. Differentiating the two is essential, given divergent treatments: SCFE requires surgical stabilization, while osteoid osteoma is optimally treated with minimally invasive RFA [9,10].

Plain radiographs are typically the first-line modality for pediatric hip pain, but computed tomography (CT) and magnetic resonance imaging (MRI) are often necessary in ambiguous cases [11,12]. MRI is effective for assessing marrow edema and soft tissue, while CT precisely identifies the nidus of osteoid osteoma [4,10,12].

## Case presentation

A 10-year-old male child with obesity (BMI 22.5 kg/m², >95th percentile for age and sex) and a history of mild childhood asthma presented with a six-month history of progressively worsening right hip pain. Initially intermittent and activity-related, the pain intensified after a football-related injury in which the patient reported a distinct “pop” in the hip. Within several weeks, the pain became constant, unresponsive to over-the-counter NSAIDs, and increasingly limited his ability to ambulate. Initial anteroposterior (AP) pelvic radiographs revealed mild irregularity of the proximal femoral epiphysis, including blurring of the physis and slight widening, as well as a subtle linear cortical density along the anterior aspect of the femoral neck, suggestive of a possible stress reaction or early physeal slip. Given these findings and the patient's obesity, SCFE was suspected, and he was referred to a pediatric emergency department for further evaluation.

**Figure 1:**
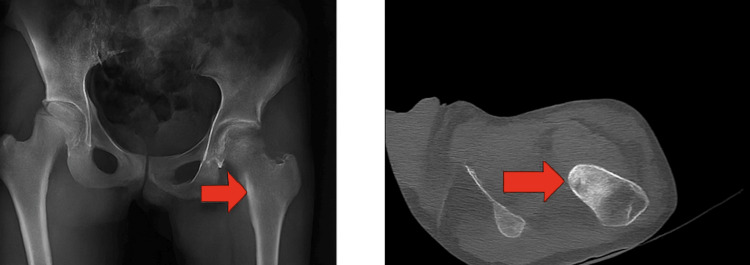
Preoperative AP Pelvic Radiograph and Axial CT Scan Showing Osteoid Osteoma AP pelvic radiograph and axial CT image demonstrate a radiolucent nidus with surrounding sclerosis in the femoral neck, typical of intra-articular osteoid osteoma. AP, anteroposterior; CT, computed tomography.

Figure 1 illustrates the preoperative AP pelvic radiograph and axial CT scan. While the radiograph suggested early SCFE, the CT revealed a radiolucent nidus with surrounding sclerosis in the femoral neck, classic for intra-articular osteoid osteoma.

On physical examination, the patient held his right leg in external rotation. Pain was elicited on both active and passive internal rotation of the hip. He exhibited an antalgic gait but had no motor or sensory deficits. There were no signs of systemic illness, such as fever, erythema, swelling, or warmth. Laboratory evaluation was notable only for a mildly elevated erythrocyte sedimentation rate (ESR) of 20 mm/h (reference: 0-10 mm/h), while his white blood cell count, C-reactive protein (CRP), and comprehensive metabolic panel were within normal limits.

MRI of the right hip demonstrated diffuse bone marrow edema in the femoral neck and adjacent soft tissues, but no evidence of epiphyseal displacement, making SCFE unlikely [8,12]. The pattern of bone marrow edema raised suspicion for an underlying intraosseous pathology. A subsequent CT scan definitively identified a 1 cm lytic lesion with a central nidus and surrounding sclerosis located in the superior aspect of the femoral neck, confirming the diagnosis of intra-articular osteoid osteoma [4,5,7].

**Figure 2:**
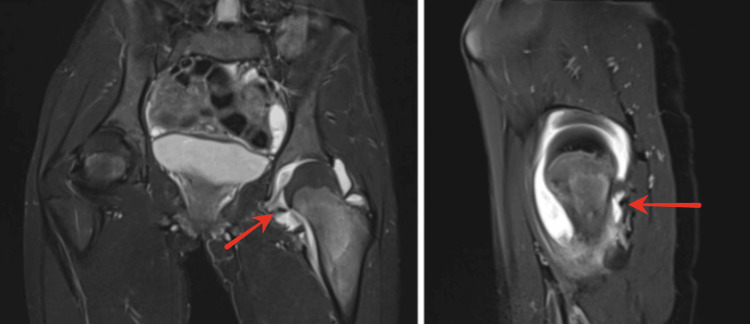
Coronal and Sagittal MRI Images Showing Bone Marrow Edema Surrounding Osteoid Osteoma Coronal and sagittal MRI images show extensive bone marrow and periarticular edema involving the femoral neck and adjacent hip joint structures, without evidence of epiphyseal displacement. MRI, magnetic resonance imaging.

Figure 2 shows the characteristic MRI findings, with coronal and sagittal views revealing extensive marrow and periarticular edema surrounding the lesion without femoral head displacement.

Hospital course and management

The patient was admitted for inpatient pain control and pre-procedural optimization. A non-weight-bearing protocol was initiated to reduce mechanical stress on the affected hip. Intravenous morphine was administered for acute pain, later supplemented with intravenous ketorolac to address inflammatory-mediated pain.

**Figure 3:**
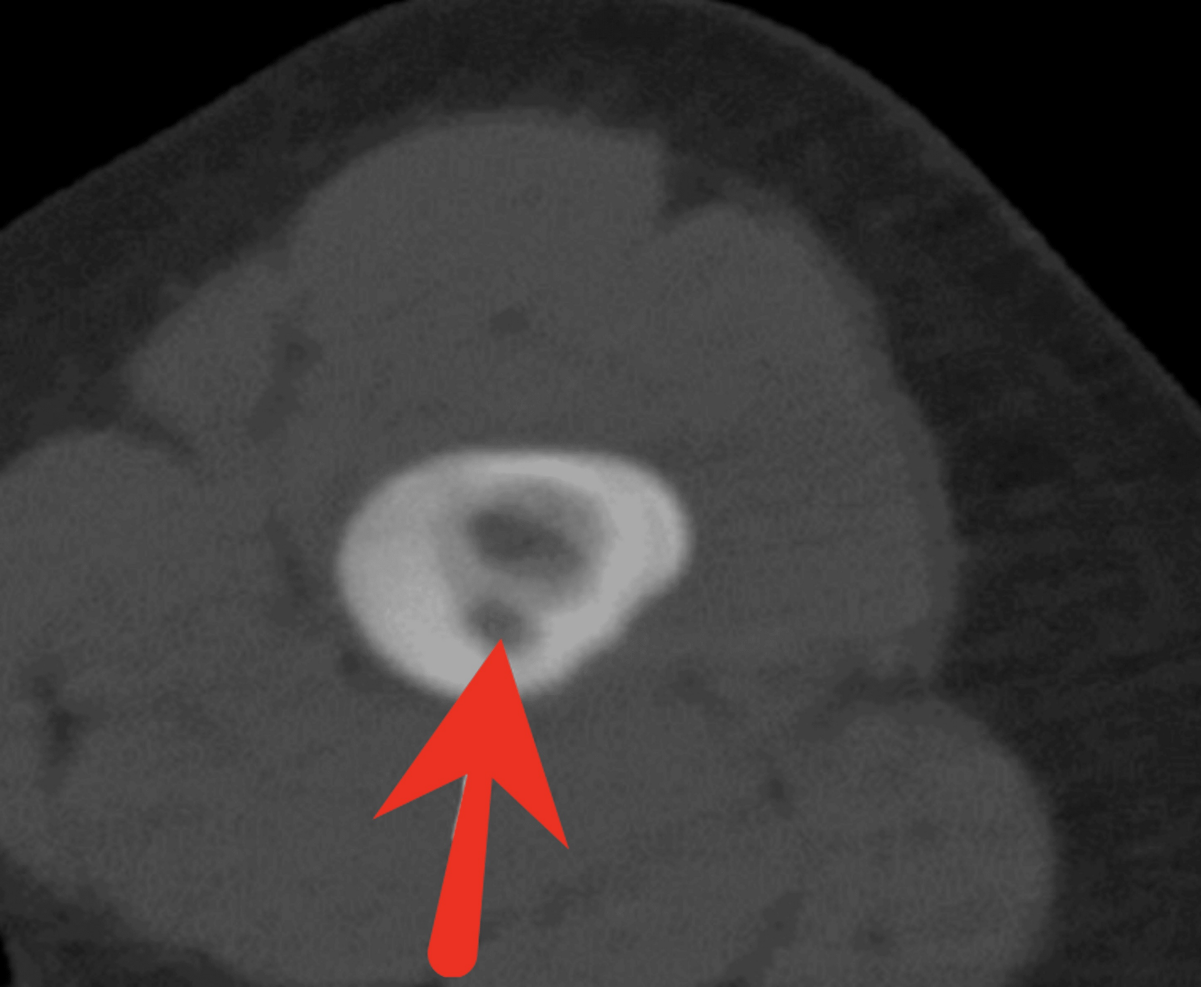
CT-Guided Radiofrequency Ablation of Osteoid Osteoma Axial intra-procedural CT image demonstrates precise placement of the radiofrequency ablation probe (red arrow) into the nidus of the osteoid osteoma located within the femoral neck. The procedure was performed under CT guidance to ensure accurate targeting of the lesion for thermal ablation. CT, computed tomography.

After interdisciplinary consultation between pediatric orthopedics and interventional radiology, the patient underwent CT-guided radiofrequency ablation (RFA) of the lesion. The procedure was enhanced with adjunctive perilesional cryotherapy and intraoperative ketorolac infusion to optimize nidus ablation and reduce postoperative pain [9,10]. Figure 3 illustrates intra-procedural CT imaging confirming accurate probe placement into the nidus.

Following the procedure, he was transitioned to oral NSAIDs and acetaminophen. Within 24 hours, he began early mobilization using an anti-gravity treadmill under physical therapy supervision. This approach, which is part of our institution’s standard postoperative rehabilitation protocol for weight-bearing joint procedures, allowed for protected ambulation while minimizing mechanical load during the early healing phase and facilitating faster functional recovery.

By hospital day 3, the patient demonstrated stable pain control, improved mobility, and was discharged with outpatient physical therapy and weight-bearing advancement instructions. His clinical course is summarized in Table 1.

**Table 1:** Clinical Timeline of Patient Management and Recovery This table outlines the patient’s clinical progression from initial presentation to long-term follow-up. It highlights key milestones in diagnosis, intervention, pain management, rehabilitation, and outcome. The case was managed using a novel, multidisciplinary approach that included CT-guided RFA, intraoperative ketorolac infusion, perilesional cryotherapy, and early mobilization using an anti-gravity treadmill. MRI, magnetic resonance imaging; RFA, radiofrequency ablation; CT, computed tomography.

Day	Clinical Milestone	Details
Day 0	Initial presentation and diagnosis	Presented to ED with progressive hip pain; MRI inconclusive; CT confirmed 1 cm intra-articular osteoid osteoma in the femoral neck.
Day 1	Inpatient management initiated	Admitted for pain control and pre-procedure optimization. Non-weight-bearing status initiated. Started on IV morphine.
Day 2	Definitive intervention	Underwent CT-guided RFA with adjunct intraoperative ketorolac infusion and localized perilesional cryotherapy.
Day 3	Post-procedure recovery and mobilization	Pain controlled with transition to oral NSAIDs and acetaminophen. Began early mobilization using anti-gravity treadmill within 24 hours post-procedure.
Week 1	Early follow-up	Outpatient follow-up: patient pain-free, normal gait resumed, continued supervised physical therapy.
Week 2	Functional recovery	Achieved full weight-bearing without assistive devices. Full range of motion restored.
Month 3	Long-term outcome	No recurrence seen on imaging. Patient discharged from orthopedic follow-up.

At one-week follow-up, the patient was pain-free with a normal gait and required no assistive devices. By week 2, he achieved full weight-bearing without limitations. At the three-month follow-up, he remained asymptomatic with full-hip range of motion and no radiologic evidence of lesion recurrence [9,10].

## Discussion

This case highlights the diagnostic challenge posed by the clinical and radiologic overlap between SCFE and osteoid osteoma, particularly when the tumor is intra-articular and located within the femoral neck [6-8]. In such cases, osteoid osteomas may present with hip pain and gait disturbances similar to SCFE, yet without the hallmark slippage of the femoral head. This diagnostic ambiguity is compounded by the fact that nidus visualization on MRI can be obscured by surrounding bone marrow edema, which may mimic the edema pattern of SCFE or even early osteomyelitis; MRI has a reported sensitivity of approximately 65% for detecting the nidus, significantly lower than that of CT [8,11].

Osteoid osteoma accounts for approximately 10-12% of all benign bone tumors, with intra-articular involvement reported in up to 10% of cases. When located in the femoral neck, these lesions may present atypically and lead to delayed or incorrect diagnosis [6].

CT remains the gold standard for identifying the nidus of osteoid osteoma and becomes essential when MRI fails to provide a definitive diagnosis [4,7,12]. This case illustrates the critical importance of a multimodal imaging strategy in evaluating complex pediatric hip pain, particularly when initial radiographs and MRI are inconclusive or misleading.

While RFA is well-established as a first-line, minimally invasive treatment for osteoid osteoma due to its high efficacy and safety profile, our case incorporates several adjunctive modalities that enhance the therapeutic outcome [9,10]. Specifically, the use of CT-guided RFA in combination with intraoperative NSAID infusion and localized perilesional cryotherapy represents a novel, multidisciplinary strategy aimed at maximizing nidus destruction, minimizing inflammation, and expediting postoperative recovery.

Equally distinctive is the rehabilitation approach employed. Early mobilization using an anti-gravity treadmill within 24 hours post-procedure allowed for protected ambulation while minimizing mechanical stress on the healing bone. This strategy may be particularly advantageous in pediatric patients, where prolonged immobility can lead to muscle atrophy, joint stiffness, and delayed return to function.

In summary, this case not only highlights the diagnostic difficulty of intra-articular osteoid osteoma mimicking SCFE but also introduces a novel therapeutic approach that integrates advanced interventional techniques with progressive rehabilitation. This combination strategy may serve as a new template for managing similar cases in pediatric orthopedic practice.

## Conclusions

This case underscores the importance of accurately distinguishing SCFE from intra-articular osteoid osteoma in pediatric patients with chronic hip pain, particularly when radiographs are equivocal. The diagnostic overlap is clinically significant, as both conditions can present with gait abnormalities and restricted motion but demand vastly different treatments. Intra-articular osteoid osteomas, unlike classic cortical lesions, may not display a visible nidus on MRI due to reactive marrow edema and joint effusion. In such cases, CT remains indispensable for identifying the central nidus. Furthermore, our approach, utilizing CT-guided RFA enhanced by perilesional cryotherapy, intraoperative ketorolac infusion, and immediate post-procedure mobilization with an anti-gravity treadmill, represents an innovative combination of established interventions that may accelerate recovery while preserving joint integrity. This case illustrates how combining interventional radiology, orthopedic input, and rehabilitative innovation can yield excellent outcomes, even in atypical intra-articular presentations. Clinicians may benefit from considering this pathway to avoid delayed diagnosis and unnecessary surgery in cases of ambiguous pediatric hip pain.
